# The snRNA-processing complex, Integrator, is required for ciliogenesis and dynein recruitment to the nuclear envelope via distinct mechanisms

**DOI:** 10.1242/bio.20136981

**Published:** 2013-11-12

**Authors:** Jeanne N. Jodoin, Mohammad Shboul, Todd R. Albrecht, Ethan Lee, Eric J. Wagner, Bruno Reversade, Laura A. Lee

**Affiliations:** 1 Department of Cell and Developmental Biology, Vanderbilt University Medical Center, Nashville, TN 37232-8240, USA; 2 Institute of Medical Biology, A*STAR, Singapore138648, Singapore; 3 Department of Biochemistry and Molecular Biology, The University of Texas Medical School at Houston, Houston, TX 77030, USA; 4 Department of Pediatrics, National University of Singapore, Singapore 119228

**Keywords:** Primary cilia, RNA processing, Dynein

## Abstract

We previously reported that the small nuclear RNA processing complex, Integrator, is required for dynein recruitment to the nuclear envelope at mitotic onset in cultured human cells. We now report an additional role for INT in ciliogenesis. Depletion of INT subunits from cultured human cells results in loss of primary cilia. We provide evidence that the requirements for INT in dynein localization and ciliogenesis are uncoupled: proteins essential for ciliogenesis are not essential for dynein recruitment to the nuclear envelope, while depletion of known regulators of perinuclear dynein has minimal effects on ciliogenesis. Taken together, our data support a model in which INT ensures proper processing of distinct pools of transcripts encoding components that independently promote perinuclear dynein enrichment and ciliogenesis.

## Introduction

Cytoplasmic dynein is a large, multimeric, minus-end-directed motor complex that associates with the dynein-activating complex, dynactin. (Holzbaur and Vallee, 1994; Kardon and Vale, 2009; Schroer, 2004). Two forms of cytoplasmic dynein exist within cells: dynein-1 and dynein-2. Dynein-1 is required for a variety of essential functions such as cargo transport along microtubules, centrosome assembly, organelle positioning, mitotic spindle positioning, and ciliogenesis, whereas dynein-2 is required for retrograde transport of cargo along primary cilia (PC) and maintenance of PC length (Palmer et al., 2011; Rajagopalan et al., 2009). Dynein complexes are subject to multiple layers of regulation, including binding of accessory proteins, phosphorylation, variations in subunit composition, and subcellular localization (Kardon and Vale, 2009).

During G2/M of cell division in multiple species, a pool of dynein anchored to the nuclear envelope (NE) facilitates nucleus-centrosome coupling, an essential step for proper mitotic spindle formation (Anderson et al., 2009; Bolhy et al., 2011; Gönczy et al., 1999; Jodoin et al., 2012; Malone et al., 2003; Robinson et al., 1999; Sitaram et al., 2012; Splinter et al., 2010; Vaisberg et al., 1993). Three components are known to be required for dynein accumulation on the NE and subsequent nucleus-centrosome coupling in human cells. The first two components, Bicaudal D2 (BICD2) and Centromere protein F (CENP-F), independently bind dynein subunits/adaptor proteins and nucleoporins to stably tether dynein complexes to the NE (Bolhy et al., 2011; Splinter et al., 2010). The third recently identified component, the small nuclear RNA (snRNA) complex, Integrator (INT), likely regulates dynein recruitment to the NE in an indirect manner distinct from that of BICD2 and CENP-F (Jodoin et al., 2013).

INT, a highly conserved nuclear complex consisting of 14 subunits, interacts with the C-terminal tail of the largest subunit of RNA-polymerase II to promote 3′-snRNA processing (Baillat et al., 2005; Chen and Wagner, 2010). These processed snRNAs play critical roles in gene expression via intron removal and further pre-mRNA processing (Matera et al., 2007). Analysis of INT has revealed that the complex must be intact to perform its RNA processing function: loss of individual INT subunits, with the exception of IntS10, leads to a nonfunctional complex (Chen et al., 2012). Various cellular functions have been ascribed to INT in cultured mammalian cells. IntS4 is required for formation of Cajal bodies (Takata et al., 2012) and IntS6 and IntS11 ensure proper differentiation of adipocytes (Otani et al., 2013). INT has additionally been reported to be required for developmental functions *in vivo*. IntS7 is essential for normal craniofacial development in both zebrafish and *C. elegans* as well as abdominal formation in *Drosophila* (Golling et al., 2002; Kamath et al., 2003; Rutkowski and Warren, 2009). In zebrafish, IntS5, IntS9, and IntS11 are required for *smad1* and *smad5* mRNA processing (Tao et al., 2009). We recently reported an essential role for INT in recruitment of dynein to the NE at G2/M (Jodoin et al., 2013).

PC are non-motile appendages that form in the majority of vertebrate cells. These structures act as sensory organelles and play essential roles in sensing and processing signals from the extracellular environment (Basten and Giles, 2013; Ko, 2012; Salisbury, 2004). To initiate primary ciliogenesis during G1, one of the two centrioles migrates to the plasma membrane, where it will dock and mature into a basal body (BB) that nucleates the PC (Kim and Dynlacht, 2013). Prior to mitotic entry, the PC is resorbed to allow for the formation of the bipolar spindle (Wang et al., 2013). Across phyla, dynein has been shown to be required for ciliogenesis (Kim and Dynlacht, 2013; Kim et al., 2011; Kong et al., 2013). Loss of dynein heavy chain, which results in decay of the complex, hinders PC formation (Draviam et al., 2006; Rajagopalan et al., 2009). Additionally, dynein is required for retrograde movement of proteins along the PC and regulation of PC length (Rajagopalan et al., 2009). While many centrosomal and interflagellar trafficking proteins are known to be critical for PC formation, more are hypothesized to exist (Graser et al., 2007; Kim and Dynlacht, 2013).

In this work, we sought to determine if INT is required for additional dynein-dependent events within cultured human cells. We herein report a role for INT in promoting ciliogenesis. We propose a model in which INT regulates dynein localization during G2/M and ciliogenesis during G1 through distinct mechanisms.

## Results and Discussion

### Individual INT subunits are required for PC formation

Given the role of INT in dynein recruitment to the NE at G2/M, we asked whether INT plays a broader role in regulating dynein-related functions. Specifically, we sought to determine if INT is required for PC formation, another dynein-dependent process. We performed siRNA-mediated down-regulation of individual INT subunits in human retinal pigment epithelial (RPE) cells and assessed PC formation. Prior to fixation and immunostaining for acetylated tubulin and γ-tubulin (to mark cilia and centrioles, respectively), the confluent monolayer of cells was subjected to serum starvation for 24 hours to stimulate PC formation.

**Fig. 1.**
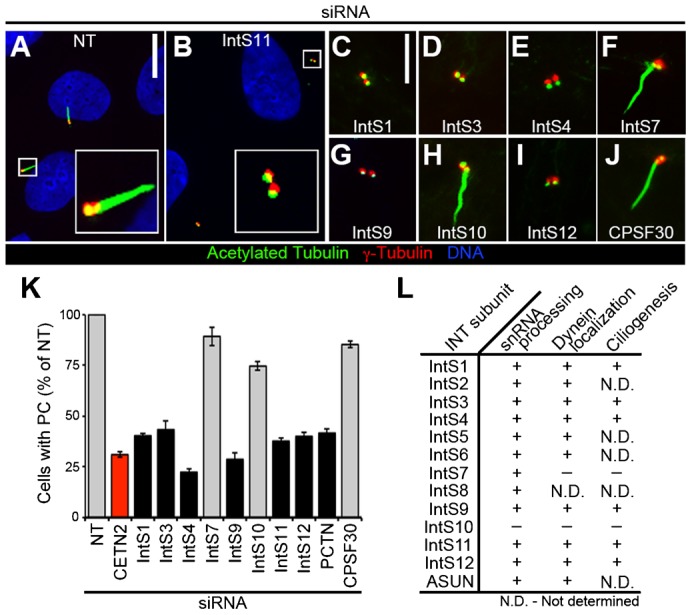
Loss of PC following INT depletion. RPE cells were transfected with siRNA, serum-starved, fixed, and stained for acetylated tubulin, γ-tubulin, and DNA. (A–J) Representative images show decreased PC formation after knockdown of most INT subunits tested. Scale bars, 10 (A,B) or 5 (C–J) µm. (K) Quantification of PC formation (normalized to NT-siRNA) in INT-depleted cells. Gray, *P*<0.0001; black, not significant (both relative to CETN2-siRNA, red). (L) Comparison of INT subunit requirements in snRNA processing (Chen et al., 2012), dynein localization, and ciliogenesis (presented herein). (+), required; (−), not required; (N.D.), not determined.

Under these conditions, the frequency of non-targeting (NT)-siRNA cells with PC ranged from ∼60–80% (data not shown). Primary ciliogenesis data are presented as the percentage of NT-siRNA cells. As a positive control, cells were depleted of Centrin-2 (CETN2), a centriolar component required for ciliogenesis (Graser et al., 2007; Salisbury, 2004). Following individual knockdown of most INT subunits tested (IntS1, 3, 4, 9, 11, or 12), we observed loss of PC to a degree comparable to that of CETN2-siRNA cells, suggesting that INT plays a critical role in PC formation (Fig. 1). We observed essentially identical results using a second independent siRNA for a subset of INT subunits, confirming that the loss of PC is not due to an off-target effect (supplementary material Fig. S1). Knockdown of IntS10, which is dispensable for both snRNA processing and dynein localization, similarly had no effect on PC formation, further confirming that this subunit is not a critical component of the INT complex. Data are conflicting for only one INT subunit, IntS7, which was previously reported to be required for snRNA processing, but not for dynein localization; we found that depletion of IntS7 had no effect on PC formation (Chen et al., 2012; Ezzeddine et al., 2011; Jodoin et al., 2013) (Fig. 1). A possible explanation for this discrepancy is that the snRNA-processing assay might be more sensitive than the assays designed to assess cytoplasmic events (perinuclear dynein accumulation and PC formation) that are presumably downstream of RNA processing. Efficient knockdown of all siRNA-targeted endogenous proteins in experiments presented herein was confirmed by immunoblotting (supplementary material Fig. S2).

To test whether the loss of PC in INT-depleted cells is specific to disruption of INT-mediated snRNA processing, and not secondary to a general disruption of RNA processing, we down-regulated Cleavage Polyadenylation Specificity Factor 30 (CPSF30) in cells and assessed PC formation. CPSF30 is a component of a complex required for the recruitment of machinery that mediates 3′-mRNA cleavage and poly(A) tail synthesis; depletion of CPSF30 leads to a deficiency, but not a complete loss, of poly(A) 3′-end formation in cells (Barabino et al., 1997). We found no significant effect on PC formation, however, in CPSF30-siRNA cells, suggesting a specific role for INT-mediated snRNA processing in PC formation (Fig. 1).

In addition to loss of PC, we observed increased centriole separation following individual knockdown of most INT subunits tested (IntS1, 3, 4, 9, 11, or 12) (results for IntS4 and IntS11 shown in supplementary material Fig. S3; data not shown for other listed subunits). This phenotype has been reported in cells depleted of a subset of ciliogenesis regulators, although a strict correlation between the separation of centrioles and loss of PC has not been observed; hence, the functional significance of this phenotype is unclear (Graser et al., 2007; Salisbury, 2004). As for PC formation, we did not observe defects in centriole coupling following IntS7 or IntS10 depletion (results for IntS10 shown in supplementary material Fig. S3; data not shown for IntS7).

We considered the possibility that the lack of PC in INT-depleted cells could be secondary to a failure of these cells to arrest in G1 upon serum starvation. We previously showed that down-regulation of individual INT subunits does not lead to any gross defects in cell-cycle phasing under normal growth conditions (Jodoin et al., 2013). To confirm that INT-depleted cells respond normally to serum starvation by arresting in G1, we performed fluorescence-activated cell sorting analysis. In contrast to INT-depleted cells growing asynchronously in 10% fetal bovine serum, INT-depleted cells arrested in G1 following serum starvation in the same manner as control cells (supplementary material Fig. S4). Taken together, these data reveal that INT is required for PC formation and centriole coupling, thereby adding to the growing list of INT-dependent cellular processes. We propose a model in which INT mediates processing of snRNA required for normal production of mRNA encoding a critical regulator of ciliogenesis.

### INT depletion does not affect a subset of proteins required for BB maturation and assembly

**Fig. 2.**
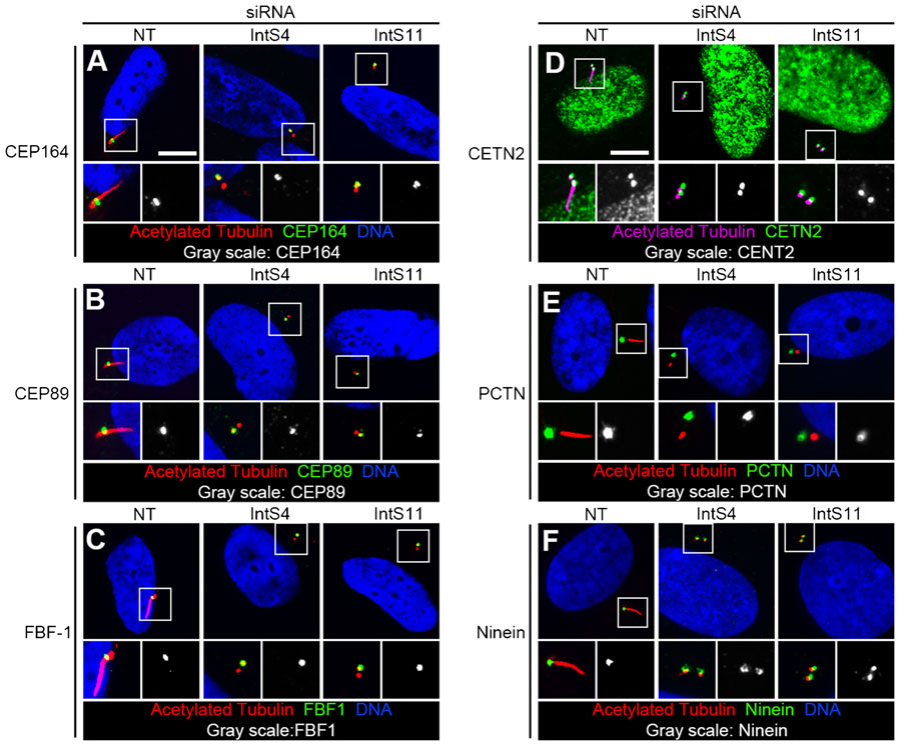
Normal BB composition following INT depletion. RPE cells were transfected with siRNA, serum-starved, fixed, and stained for acetylated tubulin, BB markers, and DNA. Representative images show normal localization of BB markers in INT-depleted cells. Higher-magnification views (bottom micrographs) of BB correspond with regions enclosed by white boxes. Scale bars, 10 µm.

We considered the possibility that loss of PC following INT depletion could result from failure of BB maturation and/or abnormal BB composition. The process of BB maturation includes assembly of the distal appendage, which projects radially from the distal end of the cilium, serving to anchor the BB and cilium to the plasma membrane. Centrosomal protein 164 (Cep164), Centrosomal protein 89 (Cep89), and Fas-binding factor 1 (FBF-1) are distal appendage components required for PC production (Sillibourne et al., 2011; Tanos et al., 2013). We observed normal localization of these proteins to the base of the PC in INT-depleted cells (Fig. 2A–C, quantified in supplementary material Fig. S5A–C). We next evaluated several core BB proteins essential for PC formation: CETN2, Pericentrin (PCTN), and Ninein (Graser et al., 2007; Salisbury, 2004). We found their localizations and intensities to be comparable in INT-depleted and control cells (Fig. 2D–F; quantified in Fig. S5D–F). We also found that γ-tubulin localized normally to BB in INT-siRNA cells (Fig. 1A–I). Taken together, these data suggest that loss of PC in INT-depleted cells is unlikely to be secondary to defective processing of transcripts encoding the studied proteins.

### PC formation is not required for dynein recruitment to the NE

We have identified two dynein-mediated processes that require INT: PC formation during G1 and dynein recruitment to the NE at G2/M (Fig. 1) (Jodoin et al., 2013). We reasoned that INT might ensure production of a single transcript encoding a common regulator of these processes or distinct transcripts that independently regulate each process. To help distinguish between these two possibilities, we sought to determine whether dynein enrichment on the NE and PC formation are interdependent or uncoupled events.

We first asked whether proteins essential for PC formation are also generally required for dynein recruitment to the NE. We performed siRNA-mediated knockdown of CETN2 and PCTN in HeLa cells and assessed dynein localization during prophase. We used the following criteria to identify prophase cells: positively immunostained for phosphorylated histone H3 (PH3) with an intact NE. HeLa cells have commonly been used to study the regulation of perinuclear dynein due to their highly enriched pool of dynein on the NE at G2/M (Bolhy et al., 2011; Jodoin et al., 2012; Jodoin et al., 2013; Splinter et al., 2010). Prior to fixation and immunostaining for dynein intermediate chain (DIC) and PH3, siRNA-treated cells were incubated with 5 µM nocodazole to stimulate recruitment of dynein-dynactin complexes and their accessory proteins to the nuclear surface. This brief nocodazole treatment has been documented to enrich for functional dynein complexes on the NE in non-G1 cells (Beswick et al., 2006; Bolhy et al., 2011; Hebbar et al., 2008; Jodoin et al., 2012; Jodoin et al., 2013; Splinter et al., 2010).

**Fig. 3.**
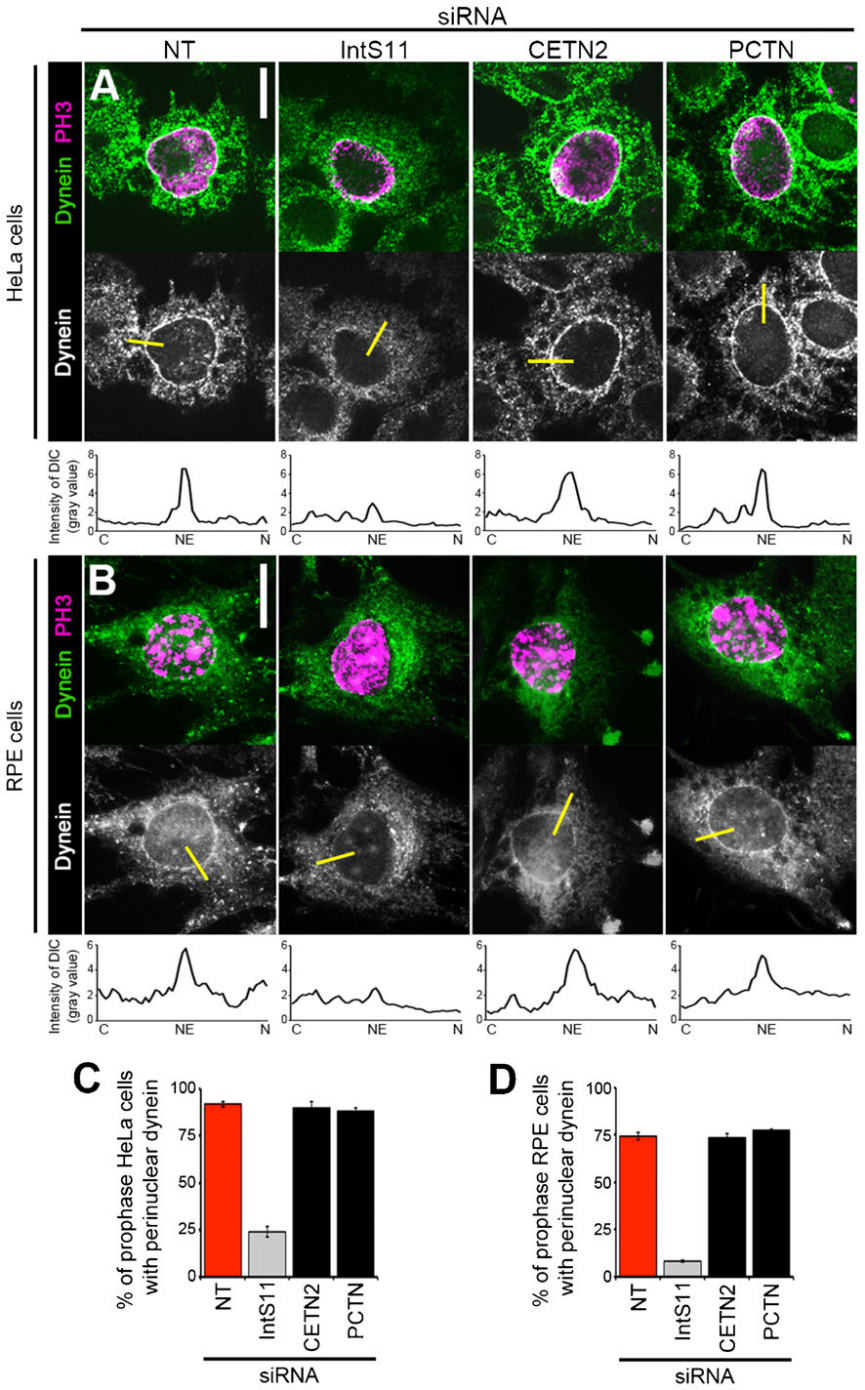
Ciliogenesis regulators are not required for perinuclear dynein recrutiment. HeLa cells (A,C) or RPE cells (B,D) were transfected with siRNA, nocodazole-treated, fixed, and stained for dynein (DIC) and PH3. (A,B) Representative images show perinuclear dynein in prophase HeLa (A) or RPE cells (B) after indicated knockdowns. Yellow bars represent line scans that span the cytoplasm (C), nuclear envelope (NE), and nucleus (N) to measure peak DIC intensity on the NE; corresponding plots are shown below each micrograph. (C,D) Quantification of perinuclear dynein in HeLa (C) or RPE (D) prophase cells (PH3-positive with intact NE). Scale bars, 10 µm. Gray, *P*<0.0001; black bar, not significant (both relative to NT-siRNA, red).

Consistent with our previous report, we observed drastic reduction of the fraction of cells with dynein accumulation on the NE following INT depletion: only 24% of IntS11-siRNA prophase cells exhibited perinuclear dynein compared to 92% of NT-siRNA prophase cells (Fig. 3A,C) (Jodoin et al., 2012). We chose to focus on IntS11 for this experiment based on its role as the catalytic subunit of the INT complex (Chen and Wagner, 2010). In contrast, the fraction of prophase cells with perinuclear dynein following depletion of other ciliogenesis regulators, CETN2 or PCTN, was comparable to that of NT-siRNA cells (90% and 88%, respectively; Fig. 3A,C). We have previously reported that this NE-anchored pool of dynein is found in RPE cells (Jodoin et al., 2012). To determine if INT is required for dynein recruitment (in addition to primary ciliogenesis) in RPE cells, we assessed perinuclear dynein following down-regulation of IntS11. Dynein accumulation on the NE was found in 77% of NT-siRNA RPE prophase cells following treatment with 10 µM nocodazole (Fig. 3B,D). Similar to our results with HeLa cells, we observed a severe reduction in the percentage of RPE prophase cells with NE-anchored dynein (down to 7%) following depletion of IntS11 (Fig. 3B,D). This finding suggests that the role for INT in promoting perinuclear dynein is conserved and is not a consequence of the transformed nature of HeLa cells. As for HeLa cells, we found that the primary ciliogenesis regulators tested were not required for perinuclear dynein in RPE cells: loss of CETN2 or PCTN recapitulated what was observed in NT-siRNA cells (with PC formation in 76% and 78%, respectively, of siRNA-treated cells; Fig. 3B,D).

As further confirmation of these findings, we quantified DIC immunofluorescence signals on the NE versus the cytoplasm and the average peak DIC intensity on the NE in both cell lines and found them greatly reduced in IntS11 cells as previously described, but unchanged in cells depleted of CETN2 or PCTN compared to NT-siRNA cells (supplementary material Fig. S6) (Jodoin et al., 2012; Jodoin et al., 2013). These data reveal that INT differs from CETN2 and PCTN, two other proteins essential for ciliogenesis, in that it has a dual function in promoting perinuclear accumulation of dynein.

### Perinuclear dynein is not required for PC formation

**Fig. 4.**
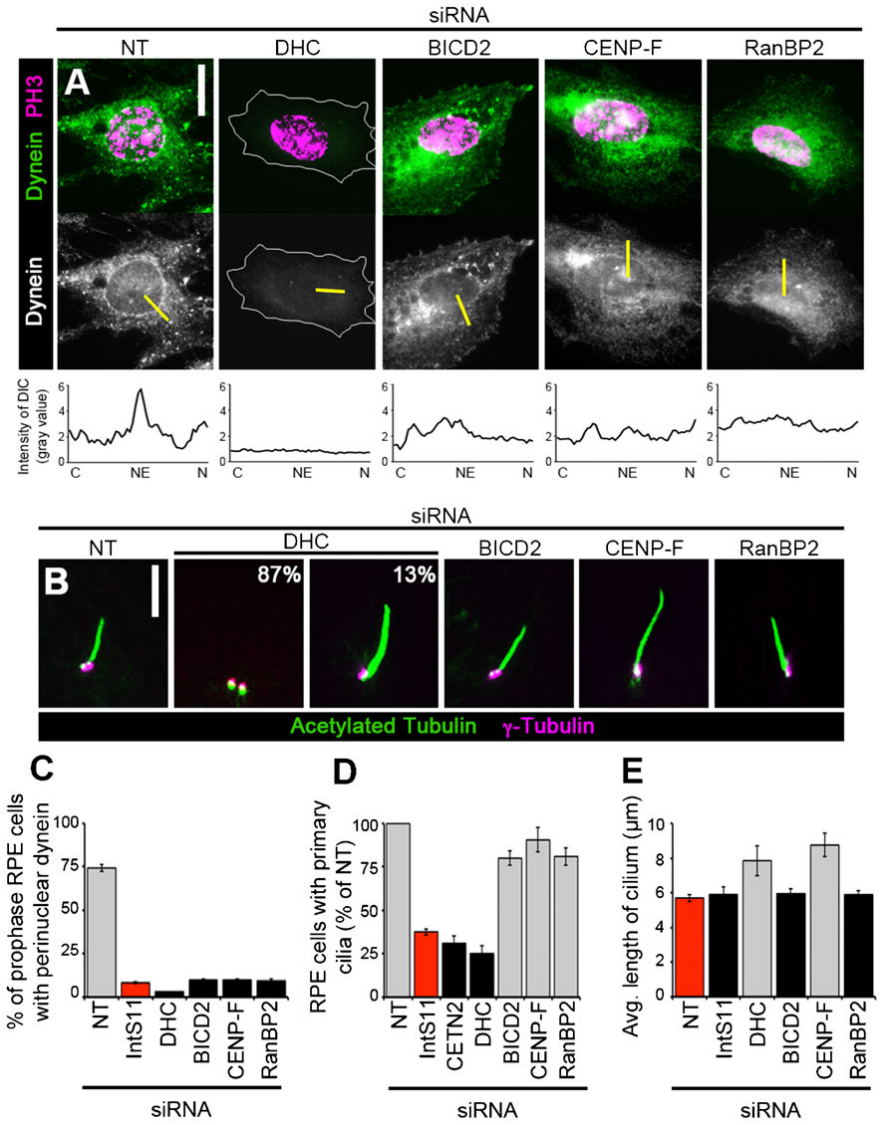
Perinuclear dynein regulators are not required for primary ciliogenesis. RPE cells were transfected with siRNA and either nocodazole-treated, fixed, and stained for dynein (DIC) and PH3 (A,C) or serum-starved, fixed, and immunostained for acetylated tubulin and γ-tubulin (B,D,E). (A) Representative images show perinuclear dynein in prophase cells (PH3-positive and intact NE) after indicated knockdowns. Yellow bars represent line scans that span the cytoplasm (C), nuclear envelope (NE), and nucleus (N) to measure peak DIC intensity on the NE; corresponding plots are shown below each micrograph. (B) Representative images show PC after indicated knockdowns. Percentages in the DHC-siRNA micrographs indicate frequency at which each phenotype (no cilium, left; elongated cilium, right) was observed. (C–E) Quantification of prophase cells with perinuclear dynein (C), PC presence (D), or average PC length (E) after indicated knockdowns. Gray, *P*<0.0001; black bar, not significant (relative to IntS11-siRNA (C,D) or NT (E), red). Scale bars, 10 µm (A) or 5 µm (B).

We next asked whether other proteins essential for perinuclear dynein accumulation are, like the INT complex, also required for primary ciliogenesis. BICD2 and CENP-F directly anchor dynein motors to the NE, whereas RanBP2 serves as the binding site for BICD2 within the nuclear pore complex; depletion of any of these proteins results in loss of perinuclear dynein in HeLa cells (Bolhy et al., 2011; Splinter et al., 2010). We confirmed that these known regulators of perinuclear dynein in HeLa cells are similarly required for NE-anchoring of dynein in cells capable of forming PC: depletion of BICD2, CENP-F, or RanBP2 in RPE cells resulted in a marked loss of perinuclear dynein compared to NT-siRNA cells (Fig. 4A,C).

We disrupted the pool of dynein anchored on the NE in RPE cells by depleting proteins required for this process and assessed PC formation. Following siRNA treatment, cells were serum-starved and evaluated for the presence of PC. Loss of BICD2, CENP-F, or RanBP2 resulted in only a slight reduction in the fraction of cells with PC (80%, 91%, and 81% of NT-siRNA cells, respectively) compared to loss of INT subunits (e.g. 38% of NT-siRNA cells for IntS11; Fig. 1K; Fig. 4B,D). Additionally we confirmed the previously reported requirement for dynein in PC formation: down-regulation of DHC resulted in the absence of PC in a majority of cells (Fig. 4B,D) (Rajagopalan et al., 2009). These data suggest that, in contrast to the INT complex, other known regulators of perinuclear dynein accumulation are not strictly required for generation of PC.

For the subset of cells with PC, we also assessed PC length in these experiments. As previously reported, we observed increased PC length following depletion of dynein when the PC was present (Fig. 4B,E) (Palmer et al., 2011). Similarly, CENP-F-siRNA cells exhibited longer PC compared to NT-siRNA cells, although BICD2 or RanBP2 depletion had no effect (Fig. 4B,E). These data suggest that CENP-F, while not required for PC formation, has a role in regulating PC length; a role for CENP-F in this process has not been previously reported to our knowledge. We speculate that CENP-F may influence PC length through its regulation of cytoplasmic dynein-2, which is essential for maintaining normal PC length (Palmer et al., 2011; Rajagopalan et al., 2009). No change in PC length, however, was observed in cells depleted of IntS11 (Fig. 1B; Fig. 4E). This finding suggests that INT promotes PC formation via a dynein-independent mechanism.

**Fig. 5.**
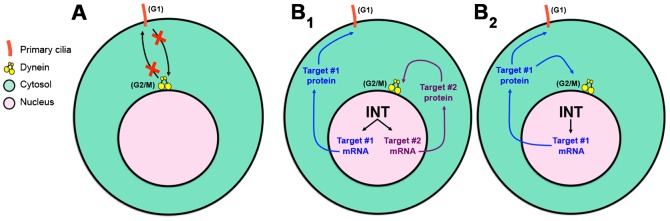
Model for independent regulation of dynein recruitment and ciliogenesis by INT. See text for details.

Taken together, the data presented herein showing that the pool of dynein anchored on the NE at the G2/M transition is not required for promoting primary ciliogenesis in G1, and vice versa, supports our hypothesis that dynein recruitment to the NE and PC formation are regulated by distinct INT-dependent mechanisms (Figs 3, 4; Fig. 5A). We propose the following model to explain how INT may regulate these two cytoplasmic events that occur at different cell-cycle stages. INT is required for proper processing of snRNAs, which in turn are required for efficient processing of mRNAs encoding at least two critical proteins that independently promote ciliogenesis in G1 and dynein recruitment to the NE at G2/M (Fig. 5B1). We cannot, however, exclude an alternative model in which INT functions through a common critical target required to mediate both of these events. In this case, INT would ensure efficient processing of transcripts encoding a single (as yet unidentified) protein that plays essential roles in promoting both ciliogenesis during G1 and perinuclear dynein accumulation at G2/M (Fig. 5B2). Further studies will be required to identify the critical target(s) of the INT complex that mediate these and other important cellular processes.

## Materials and Methods

### Cell culture, immunostaining, and microscopy

Cell lines were maintained at 37°C and 5% CO_2_ in DMEM (Life Technologies, Carlsbad, CA) containing 10% FBS, 1% L-glutamine, 100 µg/ml streptomycin, and 100 U/ml penicillin. siGENOME NT siRNA#5 (Dharmacon, Lafayette, CO) was used as negative control. siRNA oligonucleotides used herein for specific knockdowns have been previously described as follows: INT subunits (Jodoin et al., 2012), CETN2 and PCTN (Graser et al., 2007), BICD2 and RanBP2 (Splinter et al., 2010), CENP-F (Bolhy et al., 2011), and DHC (Rajagopalan et al., 2009). siRNA oligonucleotides were obtained from Dharmacon or Sigma-Aldrich (St. Louis, MO). Independent siRNA oligonucleotides used to silence IntS3 (IntS3 #2; SASI_Hs01_00063141), IntS4 (IntS4 #2; 5′-CAG CAU UGU UCU CAG AUC A-3′), IntS9 (IntS9 #2; 5′-GUG AAC UCU GCC CUU AGU A-3′), and IntS11 (IntS11 #2; SASI_Hs02_00350804) were obtained from Sigma-Aldrich. Immunoblot signals for INT subunit protein levels following siRNA treatment were quantified relative to tubulin using ImageJ.

Cells were transfected with siRNA oligonucleotides using DharmaFECT 1 transfection reagent (Dharmacon) and analyzed at 72 h post-siRNA treatment in all cases. To stimulate PC formation under conditions of serum starvation, cells at 100% confluence and at 48 h post-siRNA treatment were incubated in DMEM plus 0.5% FBS for 24 h prior to fixation. Where indicated, siRNA-treated cells in normal growth medium were incubated in 5 µg/ml (16.6 µM) nocodazole (Sigma-Aldrich) for 3 h (HeLa cells) or 10 µg/ml (33.2 µM) nocodazole for 1 h (RPE cells) prior to fixation at 72 h post-siRNA treatment to enhance perinuclear localization of dynein. Primary antibodies were used as follows: acetylated tubulin (6-11B-1, 1:500, Sigma-Aldrich), γ-Tubulin (ab16504, 1:500, Abcam, Cambridge, MA), CEP164 (NBP-77006, 1:100, Novus Biologicals, Littleton, CO), CEP89 (HPA040056, 1:100, Sigma), FBF-1 (HPA023677; 1:100, Sigma), CETN2 (N-17-R, 1:200, Santa Cruz Biotechnology, Dallas, TX), PCTN (ab4448, 1:2000, Abcam), Ninein (ab4447, 1:500, Abcam), dynein intermediate chain (74.1, 1:500, Abcam), and PH3 (Mitosis Marker, 1:1000, Millipore, Billerica, MA). Wide-field and confocal fluorescence microscopy methods were previously described (Efimov et al., 2007; Jodoin et al., 2013).

PC length (visualized by acetylated tubulin staining) was measured from base to tip using ImageJ (National Institutes of Health, Bethesda, MD); at least 100 cells were scored per condition. For determination of the percentage of cells with PC or perinuclear dynein, experiments were performed ≥3 times with ≥200 cells scored per condition. For quantification of perinuclear dynein intensity, 10 representative cells were measured per condition; for each cell, 12 line scans distributed equally around the nuclear circumference were obtained. Line scan analyses were performed using ImageJ. Line scans presented within figures are 50 pixels in length and are oriented with the cytoplasmic end of each line to the left and the intranuclear end of each line to the right. Statistical analyses of data were performed using Student's unpaired *t*-test. For bar graphs, error bars indicate s.e.m.

### Immunoblotting

Immunoblotting of cell lysates was performed as previously described (Jodoin et al., 2013). The following primary antibodies were used: c-Myc (9E10, 1:1000), β-tubulin (E7, 1:1000, Developmental Studies Hybridoma Bank, University of Iowa, Iowa City, IA), CENP-F (14C10 1D8, 1:500, Abcam), BICD2 (1:2500; gift from A. Akhmanova) (Splinter et al., 2010), dynein intermediate chain (74.1, 1:500, Santa Cruz Biotechnology), PCTN (ab4448, 1:2000, Abcam), CETN2 (N-17-R, 1:200, Santa Cruz Biotechnology), RanBP2 (ab64276, 1:1000, Abcam), IntS1, IntS4, IntS7, IntS9, IntS10, IntS11, IntS12, and CPSF30 (1:1000, Bethyl Labs, Montgomery, TX).

## Supplementary Material

Supplementary Material
