# Supplementary material for: The snRNA-processing complex, Integrator, is required for ciliogenesis and dynein recruitment to the nuclear envelope via distinct mechanisms
Source: Biol Open. 2013 Nov 12;2(12):1390–6. doi: 10.1242/bio.20136981 (PMC3863424; doi:10.1242/bio.20136981)
Supplement: Supplementary Material [file supp_2_12_1390__index.html]

The snRNA-processing complex, Integrator, is required for ciliogenesis and dynein recruitment to the nuclear envelope via distinct mechanisms — The snRNA-processing complex, Integrator, is required for ciliogenesis and dynein recruitment to the nuclear envelope via distinct mechanisms — Supplementary Material 

# The snRNA-processing complex, Integrator, is required for ciliogenesis and dynein recruitment to the nuclear envelope via distinct mechanisms

## bio.20136981 Supplementary Material

**Files in this Data Supplement:**

- Supplementary Material - Jeanne N. Jodoin et al. doi: 10.1242/bio.20136981
