# Supplementary material for: The snRNA-processing complex, Integrator, is required for ciliogenesis and dynein recruitment to the nuclear envelope via distinct mechanisms
Source: Biol Open. 2013 Nov 12;2(12):1390–6. doi: 10.1242/bio.20136981 (PMC3863424; doi:10.1242/bio.20136981)
Supplement: Supplementary Material [file supp_bio.20136981_bio.20136981-s1.pdf]

Supplementary Material

Jeanne N. Jodoin et al. doi: 10.1242/bio.20136981

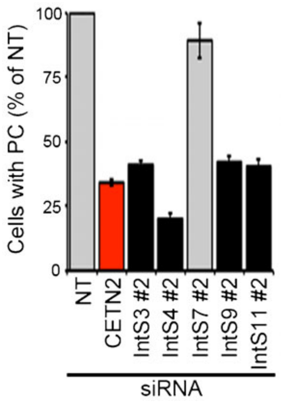

Fig. S1. Loss of PC following INT down-regulation by independent siRNA sequences. RPE cells were transfected with indicated siRNAs, serum-starved, fixed, and stained for acetylated tubulin, γ-tubulin, and DNA. Quantification of PC formation (normalized to NT-siRNA) in siRNA-depleted cells. Gray, $P<0.001$; black, not significant (relative to CETN2-siRNA, red).

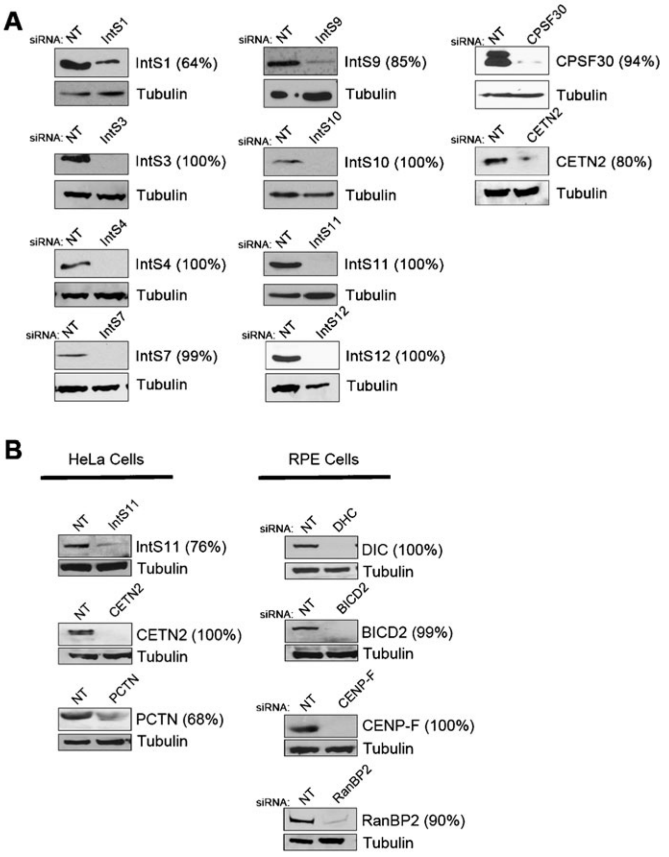

Fig. S2. Confirmation of efficient knockdown of proteins by siRNA. RPE or Hela cells were transfected with siRNA as indicated. Immunoblots of cell lysates were probed with antibodies against INT subunits, perinuclear dynein regulators, ciliogenesis regulators, or related controls corresponding to data presented in Figs 1 and 3 (A) or Figs 3 and 4 (B). Depletion of each protein targeted by siRNA was confirmed. Tubulin was used as loading control. Percent of protein depletion following targeted knock down indicated next to each antibody.

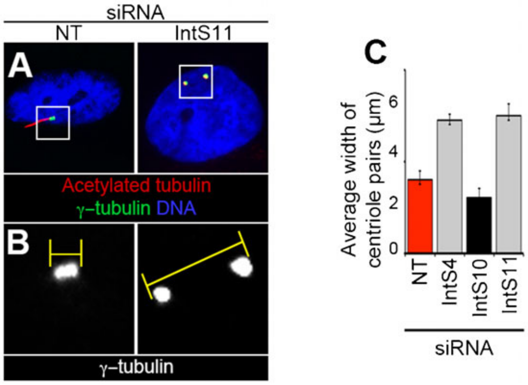

Fig. S3. Increased frequency and degree of centriole pair separation following loss of INT activity. RPE cells were transfected with siRNA as indicated. Following siRNA treatment, PC formation was stimulated by serum starvation. After fixation, cells were stained for acetylated tubulin, g-tubulin, and DNA. (A) Separation of centriole pairs was observed following INT subunit-siRNA treatment. (B) Higher magnification of region enclosed within white box shows scoring system used to measure distance between centriole pairs (yellow bars). (C) Quantification of the average distance between centriole pairs following indicated siRNA treatment. Gray bars, $P<0.0001$; black, not significant (both relative to NT-siRNA, red). Distance between the outer borders of basal body pairs (marked by g-tubulin staining) was measured using ImageJ. Basal body pairs in at least 100 cells were scored per condition.

Biology Open

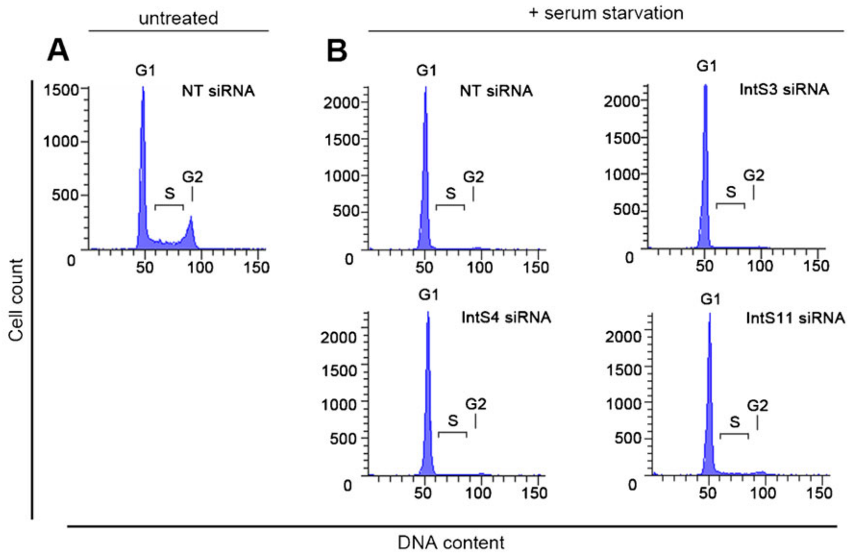

Fig. S4. Confirmation of G1 arrest in serum-starved RPE cells following INT subunit knockdown. RPE cells were transfected with siRNA as indicated, serum-starved, fixed, and stained with propidium iodide. DNA content was analyzed by FACS as previously described (Jodoin et al., 2013). (A) Cell-cycle profile of NT-siRNA cells grown under normal medium conditions (plus 10% FBS). (B) Cell-cycle profiles of NT-siRNA and INT-depleted cells following serum starvation revealed a similar degree of G1 arrest.

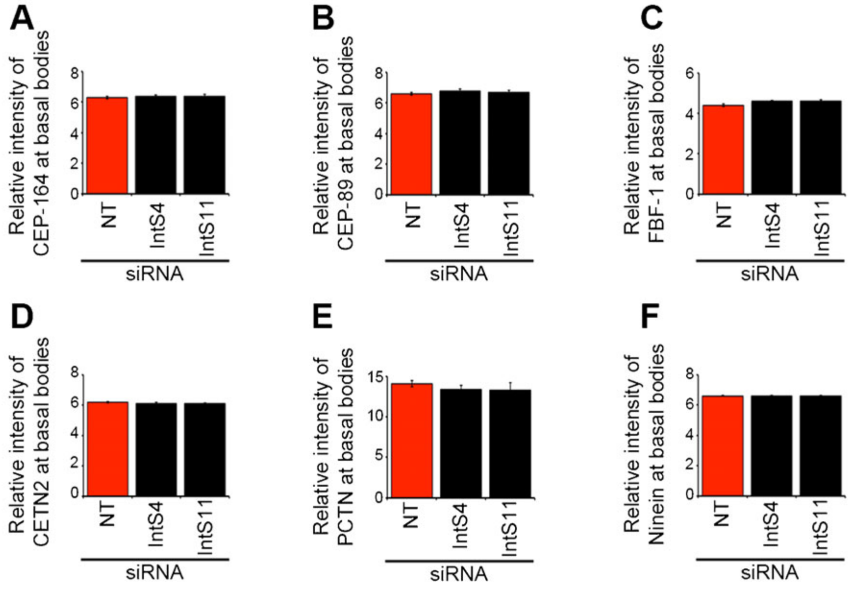

Fig. S5. Characterization of basal body markers. Quantification of distal appendage (A–C) and basal body (D–F) signals for indicated markers (normalized to signals for acetylated tubulin). No significant changes in intensity were observed following depletion of individual INT subunits. Intensity of antibody signals on basal bodies was determined using ImageJ. Basal bodies in at least 100 cells were scored per condition. Black bar, not significant (relative to NT-siRNA, red).

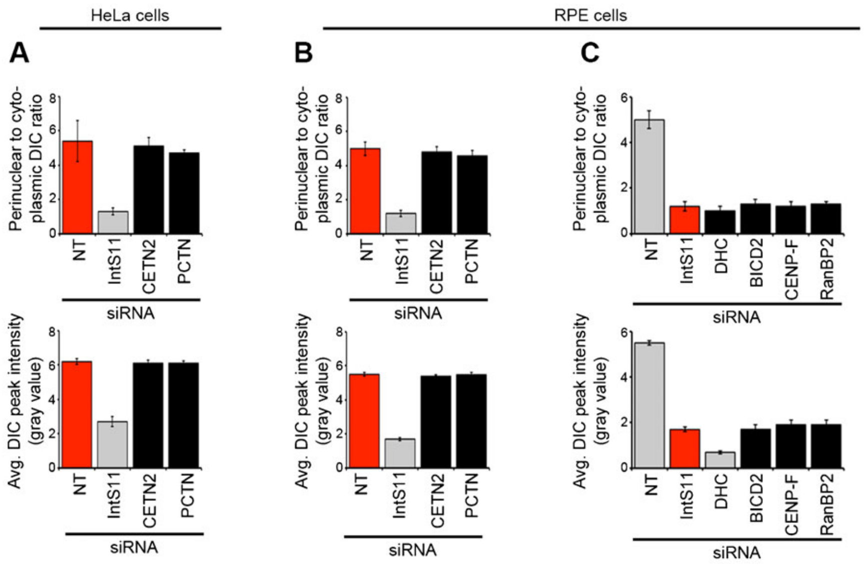

Fig. S6. Quantification of perinuclear dynein following knockdown of ciliogenesis regulators. Bar graphs correspond to representative images of immunostained HeLa (A) or RPE (B,C) cells presented in Fig. 3A,B and Fig. 4A. Line scans were drawn from the cytoplasm to inside the nucleus. Ratios of the intensity of the dynein signal on the NE to the cytoplasm (top) and average peak intensities of perinuclear dynein (bottom) are shown. Samples with $P<0.0001$ are shown in gray; black, not significant (relative to NT-siRNA (A,B) or IntS11-siRNA (C), red).

Biology Open
